# Grid cells, border cells, and discrete complex analysis

**DOI:** 10.3389/fncom.2023.1242300

**Published:** 2023-10-10

**Authors:** Yuri Dabaghian

**Affiliations:** Department of Neurology, The University of Texas, McGovern Medical Center at Houston, Houston, TX, United States

**Keywords:** grid cells, border cells, percolation, discrete complex analysis, learning and memory, hippocampo-cortical network

## Abstract

We propose a mechanism enabling the appearance of border cells—neurons firing at the boundaries of the navigated enclosures. The approach is based on the recent discovery of discrete complex analysis on a triangular lattice, which allows constructing discrete epitomes of complex-analytic functions and making use of their inherent ability to attain maximal values at the boundaries of generic lattice domains. As it turns out, certain elements of the discrete-complex framework readily appear in the oscillatory models of grid cells. We demonstrate that these models can extend further, producing cells that increase their activity toward the frontiers of the navigated environments. We also construct a network model of neurons with border-bound firing that conforms with the oscillatory models.

## 1. Introduction and motivation

**Figure 1:**
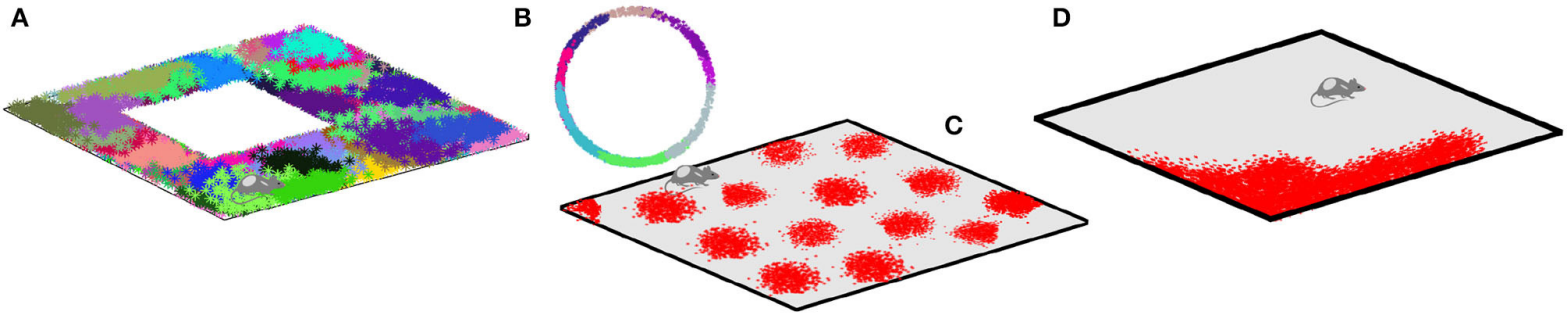
Spatial cells. **(A)** Spikes produced by place cells (dots of different colors) form distinct spatial clusters in the navigated environment, which highlight the preferred spiking domains—place fields. **(B)** Head direction cells fire when the animals' head is oriented at a particular angle with respect to cardinal directions, thus producing spike clusters in the circular space of planar directions. **(C)** Spiking domains of the grid cells form a triangular lattice that tiles the ambient space. **(D)** Boundary cells produce spikes along the border of the navigated enclosure.

Spiking activity of spatially tuned neurons is believed to enable spatial cognition (Moser et al., 2008; Grieves and Jeffery, 2017; Derdikman and Moser, 2010). For example, rodent's*place cells*^1^ that fire in specific locations produce a qualitative map of the explored environment (Gothard et al., 1996; Alvernhe et al., 2012; Dabaghian et al., 2014; Wu and Foster, 2014; Rueckemann et al., 2021); *head direction* cells that fire each at its preferred orientation of the animals' head contribute directional information (Taube, 1998; Dabaghian, 2022; Valerio and Taube, 2012); the *grid cells* that fire near vertexes of a planar triangular lattice are believed to provide a metric scale (Hafting et al., 2005; Moser et al., 2008) and the *border cells* highlight the boundaries of the navigated enclosures (Lever et al., 2009; Barryet al., 2006; Solstad et al., 2008) (Figure 1).

A number of theoretical models aim to explain the machinery producing these spiking profiles, by exploiting suitable mathematical phenomena, e.g., attractor network dynamics (Tsodyks, 2005; Rolls, 2007; Colgin et al., 2010; Bassett et al., 2018; Giocomo et al., 2011), specific network architectures (Colgin et al., 2010; Bush et al., 2014; Cheng and Frank, 2011; Solstad et al., 2006), the hexagonal symmetry of closely packed planar discs (Fuhs and Touretzky, 2006), constructive interference of symmetrically propagating waves (Burgess et al., 2007; Hasselmo et al., 2007; Burgess, 2008; Burgess and O'Keefe, 2011), and so forth. In contrast, the ability of border cells to identify the frontiers of the explored environments was heretofore explained heuristically, as a certain “responsiveness” these neurons to the walls of the navigated arenas, achieved, conceivably, by integrating proprioceptive and sensory inputs (O'Keefe and Burgess, 1996; Burgess and Hartley, 2002; Raudies and Hasselmo, 2012; Hartley et al., 2000; Burgess et al., 2000). However, since border cells are anatomically removed from sensory pathways, it is possible that their spiking may be produced through autonomous network mechanisms, rather than induced by external driving. From a computational perspective, such mechanisms may also hinge on a mathematical phenomenon that highlights the perimeters of spatial regions, a well-known example of which is the *maximum principle*—the ability of certain functions, e.g., harmonic and complex-analytic functions, to attain maximal values at the boundaries of their domains (Marsden and Hoffman, 1999).

The following study is motivated by a recent series of publications (Novikov and Dynnikov, 2003; Novikov, 2004, 2011; Dynnikov, 2015), which show that two-dimensional (2*D*) triangular lattices allow constructing a discrete counterpart of the Complex Analysis and defining real-valued, discrete epitomes of complex-analytic functions that obey the maximum principle. As it turns out, these structures allow modeling border cell activity, as discussed below.

The paper is organized as follows. Several key ideas of Discrete Complex Analysis (DCA) are outlined in Section 2, following the exposition given in Novikov and Dynnikov (2003); Novikov (2004, 2011); Dynnikov (2015). Section 3 discusses certain connections between elements of DCA and oscillatory interference models of grid cells (Burgess et al., 2007; Hasselmo et al., 2007; Burgess, 2008; Burgess and O'Keefe, 2011), and offers a generalized framework for expanding these models to include border cell spiking patterns. In Section 4, elements of DCA are implemented in a schematic network model that produces border cell firing responses through endogenous activity, without using external parameters, such as animal's speed or location. The results are briefly discussed in Section 5.

## 2. Approach

**1. Discrete complex analysis**. Standard theory of complex variables is a calculus over complex numbers *z* = *x* + *iy* and their conjugates, $\bar{z}=x-iy$, where *x* and *y* are the Cartesian coordinates in a Euclidean plane and *i* is the imaginary unit, *i*^2^ = −1 (Marsden and Hoffman, 1999). A generic complex function depends on both *z* and $\bar{z}$; however, the main objects of the theory are the *analytic* (also called *holomorphic*) functions that depend only on *z*, *f* = *f*(*z*), and their *anti-analytic* (*anti-holomorphic*) counterparts, that depend only on $\bar{z}$, $f=f\left(\bar{z}\right)$. The defining property of these functions is that their derivatives over the “missing” variable vanish,


$$\begin{array}{l} \frac{\partial f}{\partial \bar{z}}=\left(\frac{\partial }{\partial x}+i\frac{\partial }{\partial y}\right)f=0,\text{ for analytic functions},\\ \frac{\partial f}{\partial \bar{z}}=\left(\frac{\partial }{\partial x}-i\frac{\partial }{\partial y}\right)f=0,\text{ for anti-analytic functions}. \end{array}$$

The Cauchy operator and its conjugate used above,


$$\partial \equiv \frac{\partial }{\partial x}+i\frac{\partial }{\partial y},\bar{\partial }\equiv \frac{\partial }{\partial x}-i\frac{\partial }{\partial y},$$

play key roles not only in complex analysis but also in geometry and applications. One of their properties is that they factorize the 2*D* Laplace operator, or the *Laplacian*,


(1) $$\Delta \equiv \frac{\partial^{2}}{\partial x^{2}}+\frac{\partial^{2}}{\partial y^{2}}=\left(\frac{\partial }{\partial x}+i\frac{\partial }{\partial y}\right)\left(\frac{\partial }{\partial x}-i\frac{\partial }{\partial y}\right)\equiv \partial \bar{\partial }.$$

The factorization (1) is unique and necessarily involves complex numbers—think of the decomposition *x*^2^ + *y*^2^ = (*x* + *iy*)(*x* − *iy*) that is commonly used to motivate the transition from real to complex variables. Correspondingly, the phenomenon (1) takes place only on spaces that admit complex structure—orientable 2*D* surfaces. Furthermore, the factorization (1) can serve as a vantage point for defining the Cauchy operator and its conjugate: if a Laplacian admits the decomposition (1) in suitable coordinates, then the resulting curvilinear first-order operators $\bar{\partial }$ and ∂ will be the Cauchy operators of a complex-analytic structure on the corresponding manifolds.

A remarkable observation made in (Novikov and Dynnikov, 2003; Novikov, 2004, 2011; Dynnikov, 2015) is that the *discrete* Laplace operator on a 2*D* triangular lattice also is factorizable. Indeed, a generic discrete Laplacian on a graph or a lattice acts on the vertex-valued functions *f*(*v*) as


(2) $$\Delta f(v)=\sum_{v^{\prime }}f(v^{\prime })-\rho_{v}f(v),$$

where the summation goes over all vertexes *v*′ linked to *v*, and *ρ*_*v*_ is the valency of *v* (Sarnak, 1990; Godsil and Royle, 2001; Belkin et al., 2008). On a triangular lattice with vertexes marked by two integer indexes *m* and *n*, the Laplacian (2) becomes


(3) Δf=f(m+1,n+1)+f(m+1,n)+…+f(m−1,n+1)−6f(m,n).

**Figure 2:**
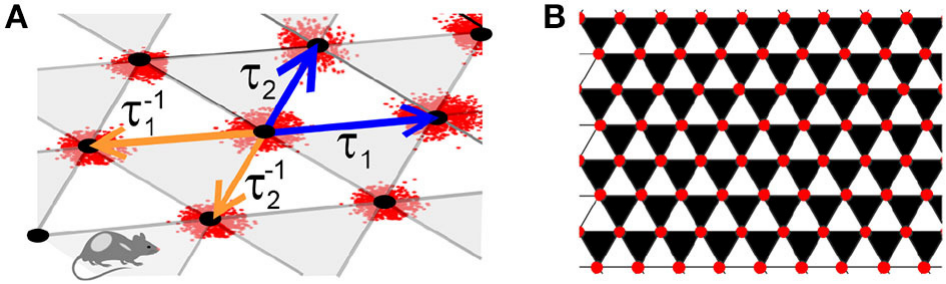
Lattice. **(A)** The operators *τ*_1_ and *τ*_2_ (blue arrows), shift the argument of the lattice function forward along the basis directions, from (*m, n*) to (*m*+1, *n*) and (*m, n*+1), respectively. The inverse operators, $\tau_{1}^{-1}$ and $\tau_{2}^{-1}$ (orange arrows), shift the argument backwards, correspondingly to (*m*−1, *n*) and (*m, n*−1). **(B)** The backwards shifts $\tau_{1}^{-1}$ and $\tau_{2}^{-1}$ support the “black" triangles and forward shifts *τ*_1_ and *τ*_2_ span the complimentary set of “white" triangles. If a function satisfies the discrete-analyticity condition (8a), then its values over the black triangles vanish.

To obtain the required decomposition, let us define the operators *τ*_1_ and *τ*_2_ that shift the arguments of the vertexes functions,


(4a) $$\tau_{1}f(m,n)=f(m+1,n),$$

(4b) $$\tau_{2}f(m,n)=f(m,n+1).$$

as shown on Figure 2A. In terms of *τ*_1_ and *τ*_2_, the sum (3) becomes


(5) $$\Delta_{L}=\tau_{1}+\tau_{2}+\tau_{1}^{-1}+\tau_{2}^{-1}+\tau_{2}\tau_{1}^{-1}+\tau_{1}\tau_{2}^{-1}-6,$$

and factorizes into the product of two first-order operators


(6a) $$Q=1+\tau_{1}+\tau_{2},$$

(6b) $$\bar{Q}=1+\tau_{1}^{-1}+\tau_{2}^{-1},$$

with an extra constant term,


(7) $$\Delta_{L}=Q\bar{Q}-9.$$

As shown in Novikov and Dynnikov (2003); Novikov (2004, 2011); Dynnikov (2015), this decomposition induces a DCA, in which the operator $\bar{Q}$ plays the role of the complex-conjugate derivative $\bar{\partial }$. One can thus define the discrete-analytic lattice functions, *f*(*m, n*), as the ones that satisfy the relationship


(8a) $$\bar{Q}f(m,n)=f(m,n)+f(m-1,n)+f(m,n-1)=0.$$

The *Q*-operator then acts as the discrete-analytic derivative,


(8b) Qf(m,n)=f(m,n)+f(m+1,n)+f(m,n+1).

Geometrically, equations (8) can be illustrated by partitioning the lattice *V* with “black" and “white" triangles, in which each white triangle, ▵, shares sides with three black triangles, ▾, and vice versa (Figure 2B). According to (8a), the discrete analytic functions vanish over all the black triangles, which may be viewed as the lattice analogue of “*z*-but-not-$\bar{z}$" dependence of the conventional complex-analytic functions.

**2. Properties of the discrete-analytic functions** largely parallel the familiar properties of their continuous counterparts, including the maximal principle that is used below to model the border cell spiking activity. However, there are also a few differences, the most striking of which is that the discrete-analytic functions are *real-valued*: indeed, the equation (8a) does not involve imaginary numbers and possesses real-valued solutions (Novikov and Dynnikov, 2003; Novikov, 2004, 2011; Dynnikov, 2015). Thus, the discrete complex analysis is a real-valued combinatorial framework that may be implemented through neuronal computations.^2^

Another peculiarity is that DCA redefines the notion of a constant. Indeed, the constants *c* of the standard calculi are nullified by the derivatives, $\partial c=\bar{\partial }c=0$. However, a quantity that assumes constant values on all vertexes, *f*(*m, n*) = *c*, is not nullified, but tripled by discrete derivative operators, $Qc=\bar{Q}c=3c$. Hence, discrete-analytic constants *h* must be derived from the equations


(9) $$\bar{Q}h=Qh=0.$$

**Figure 3:**
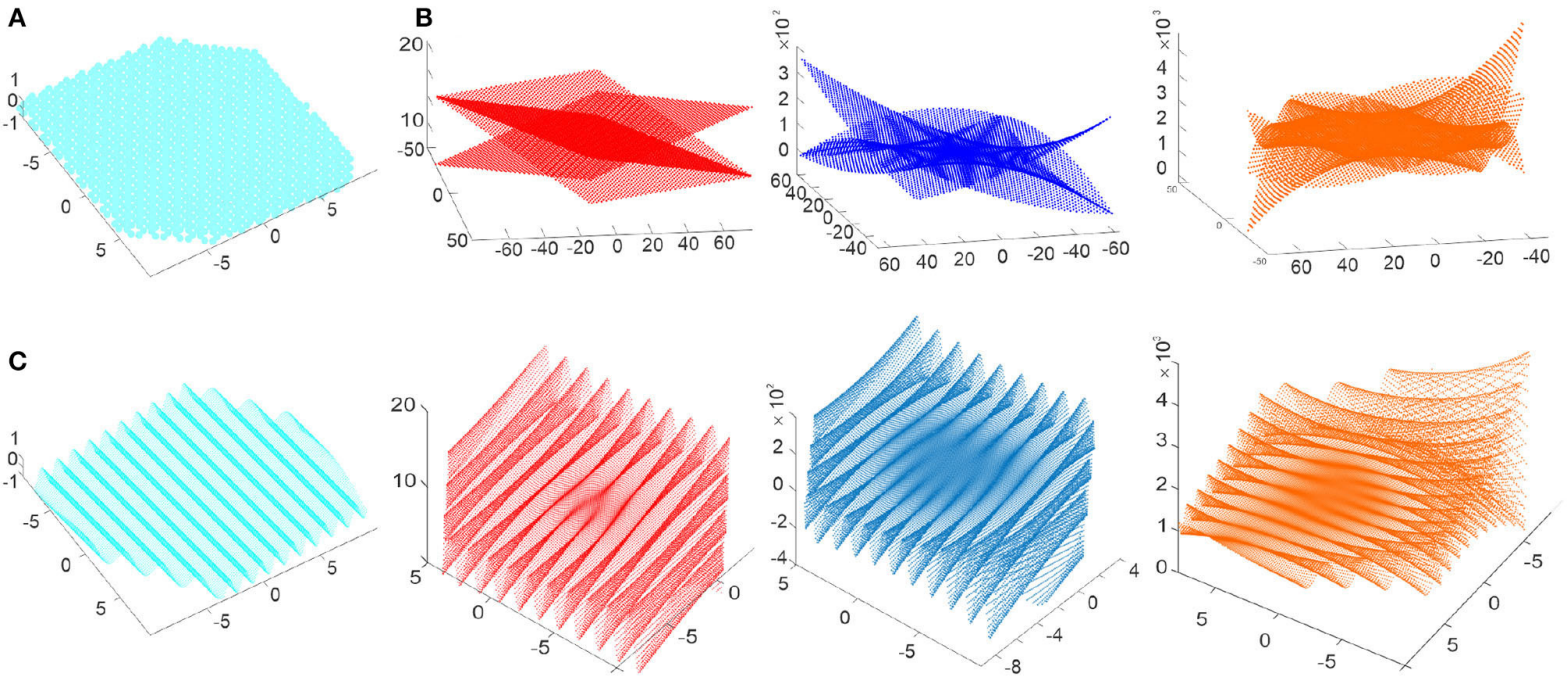
Discrete-analytic polynomials. **(A)** 0*th* order polynomials are the holomorphic constants that assume a small set of discrete values *h*_1_ = {−0.5, 1} and $h_{2}=\left\{\pm \sqrt{3}/2,0\right\}$. **(B)** The discrete-holomorphic polynomials *P*_1_(*m, n*), *P*_2_(*m, n*) and *P*_3_(*m, n*) grow outwards (linearly, parabolically and cubically) as the lattice indexes increase. **(C)** The spatially-refined discrete polynomials produce undulatory shapes scaffolded by their discrete counterparts: shown are the undulating holomorphic wave *h*_1_(*x, y*) and the polynomials *P*_1_(*x*_1_, *x*_2_), *P*_2_(*x*_1_, *x*_2_) and *P*_3_(*x*_1_, *x*_2_) that grow toward the boundary of the enclosed Euclidean domain.

Somewhat surprisingly, the basic solutions of (9) have the form


(10) $$h(\delta )=\cos\frac{2\pi }{3}(n+2m+\delta ),$$

where δ is a phase parameter (Figure 3A). Formula (10) can be viewed as a discrete analogue of the complex phase *e*^*iδ*^; the “prime" constants 1 and *i* then correspond to


(11a) $$h_{1}=\cos\frac{2\pi }{3}(n+2m),$$

(11b) $$h_{2}=\sin\frac{2\pi }{3}(n+2m).$$

Note that, in contrast with their familiar counterparts, the “constants" (10) and (11) alternate from vertex to vertex, assuming a few discrete values, *h*_1_ = {−0.5, 1} and $h_{2}=\left\{\pm \sqrt{3}/2,0\right\}$.

The third distinct property concerns Taylor-expansions: in contrast with the continuous case, a generic discrete-holomorphic function *f*(*m, n*) over a finite lattice domain can be represented exactly by finite series, i.e., one can write


$$f(m,n)=U(m,n)h_{1}+W(m,n)h_{2},$$

where *U*(*m, n*) and *W*(*m, n*) are polynomials. The order of such polynomials generally grows with the size of the lattice domain, which allows keeping the above expansion exact.

Explicit examples of the first, second and third-order discrete-analytic polynomials are


(12a) $$P_{1}=-\frac{\sqrt{3}}{2}(m+n)h_{1}+\frac{1}{2}(n-m)h_{2},$$

(12b) $$\begin{array}{l} P_{2}=(m-n)(3(n+m)-1)h_{1}\\ \;-\sqrt{3}({\left(m+n\right)}^{2}+2mn-3(n+m))h_{2}. \end{array}$$

(12c) $$\begin{array}{l} P_{3}=(m-n)((m+2n)(2m+n)-2(3(m+n)-1))h_{1}\\ \;+\sqrt{3}(6(m+n)-2mn-4{\left(m+n\right)}^{2}+3mn(m+n))h_{2}, \end{array}$$

illustrated on Figures 3B, C. It can be verified by direct substitution^3^ that the operator $\bar{Q}$ nullifies each polynomial, whereas *Q* lowers their order, *QP*_1_ = *h*_1_, *QP*_2_ ∝ *P*_1_ and *QP*_3_ ∝ *P*_2_, just as $\bar{\partial }$ would nullify polynomials of *z*, and ∂ would lower their order, ∂*p*_*r*_(*z*) ∝ *p*_*r*−1_(*z*). In general, there are 2(*r* + 1) basic discrete-analytic polynomials of order *r*, which corresponds to 2(*r* + 1) basic complex *rth*-order complex polynomials (Novikov and Dynnikov, 2003; Novikov, 2004, 2011; Dynnikov, 2015).

**3. Spatial fine-graining**. Discrete functions defined over the lattice vertexes give rise to finer-grained spatial structures. Given two basis vectors


(13) $${\vec{e}}_{1}^{\;g}=a_{g}(1,0),\;{\vec{e}}_{2}^{\;g}=a_{g}\left(1/2,\sqrt{3}/2\right),$$

in the Euclidean plane, consider a lattice generated by integer translations,


(14) $$V_{g}=\{v_{m,n}^{g}=m{\vec{e}}_{1}^{\;g}+n{\vec{e}}_{2}^{\;g},m,n\in \mathbb{Z}\}.$$

Such embedding allows extending the discrete argument of a vertex function, *f*(*m, n*), to a function of Euclidean coordinates, *f*(*x*_1_, *x*_2_), by replacing the integer arguments (*m, n*) with pairs of reals (*x*_1_, *x*_2_). For example, the discrete-holomorphic constant (11a) yields a continuous “holomorphic wave” with wavelength ∝*a*_*g*_,


(15) $$\cos\frac{2\pi }{3}(2m+n)\to \cos\frac{2\pi }{3a_{g}}(2x_{1}+x_{2}),$$

propagating in the direction ${\vec{e}}_{1}$ (Figure 3C). Conversely, using


$$x_{1}=a_{g}m+\delta_{1},\;x_{2}=a_{g}n+\delta_{2}$$

in the real-valued functions with sufficiently low spatial frequency (<2π/*a*_*g*_) restores the dependence upon the lattice indexes and produces a continuous phase δ that contains fractional remainders,


(16) $$\cos\frac{2\pi }{3a_{g}}(2x_{1}+x_{2})\to \cos\frac{2\pi }{3}(n+2m+\delta ).$$

The latter form allows acting with the operators *Q* and $\bar{Q}$ on the regular coordinate functions and placing the results into the context of DCA.

## 3. Oscillatory grid cell models

Surprisingly, discrete-analytic structures are manifested in the existing models of grid cell activity, e.g., in the oscillatory interference models that derive the observed grid field patterns from the dynamics of the membrane potential,


(17) $$\mu_{g}(t)=\prod_{k=1}^{3}\left(\cos(\omega t)+\cos\left(\omega t+\beta \int_{0}^{t}\langle {\vec{l}}_{k}^{\;g}\cdot \vec{v}\rangle dt\right)\right){|}_{\theta }.$$

**Figure 4:**
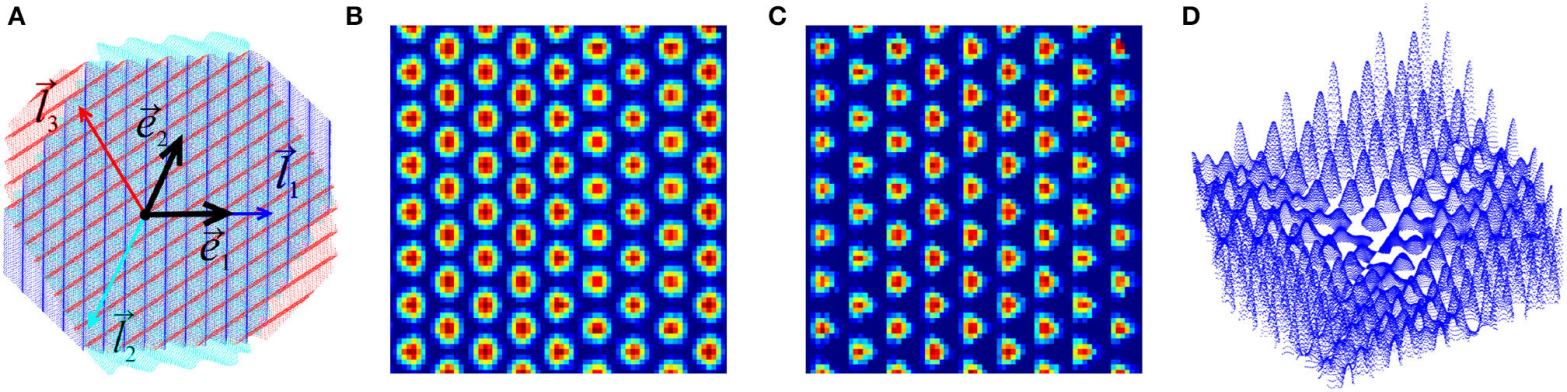
Oscillatory interference model. **(A)** Superposition of three discrete-holomorphic waves propagating in three symmetric directions specified by the three wave vectors ${\vec{l}}_{1}$, ${\vec{l}}_{2}$, and ${\vec{l}}_{3}$. The basis lattice directions ${\vec{e}}_{1}$ and ${\vec{e}}_{2}$ are shown in black. Constructive interference occurs at the vertexes of a triangular lattice, highlighted by the amplitude (18). **(B)** The grid cell firing amplitude, *A*_*g*_, formula (19), reproduces the familiar grid cell layout. **(C)** The complementary “conjugate" amplitude, Ã_*g*_ = sin*φ*_1_ sin*φ*_2_ sin*φ*_3_. **(D)** The second order discrete-holomorphic *grid-polynomial* (21b), defined over the grid fields, compare with the third panel on Figure 3C.

Here *t* is time, *β* is a scale parameter, ${\vec{l}}_{1}^{g}$, ${\vec{l}}_{2}^{g}$ and ${\vec{l}}_{3}^{g}$ are the three symmetric wave vectors, $\vec{v}\left(t\right)$ is the velocity, and ω ≈ 8 Hz is the mean frequency of the synchronized extracellular field's oscillations. The index “θ" refers to the firing threshold (Burgess et al., 2007; Hasselmo et al., 2007; Burgess, 2008; Burgess and O'Keefe, 2011). Due to the symmetry, the waves interfere constructively at the vertexes of a triangular lattice with basis vectors ${\vec{e}}_{1}^{g}={\vec{l}}_{1}^{g}$ and ${\vec{e}}_{2}^{g}=-{\vec{l}}_{2}^{g}$, centered at the firing fields^4^ (Figures 1C, 4A).

To link μ_*g*_(*t*) to DCA, let us rewrite the time integrals in (17) as integrals along the trajectory,


$$\begin{array}{r} \mu_{g}(t)=\prod_{k=1}^{3}\left(\cos\omega t+\cos\left(\omega t+\frac{8\pi }{3a_{g}}\langle {\vec{l}}_{k}^{\;g}\cdot \int_{\gamma }d\vec{r}\rangle \right)\right){|}_{\theta }\\ =A_{g}(\vec{r})\prod_{k=1}^{3}\cos\left(\omega t+\varphi_{k}^{g}(\vec{r})\right){|}_{\theta }, \end{array}$$

where $\vec{r}$ is the position vector, $\dot{\vec{r}}=\vec{v}$, $\varphi_{k}^{g}$ are the oscillatory phases and 8π/3*a*_*g*_ = *β*. The time-independent factor defines the spatial amplitude of the membrane potential,


(18) $$A_{g}(\vec{r})=\prod_{k=1}^{3}\cos\frac{4\pi }{3a_{g}}\left({\vec{l}}_{k}^{\;g}\cdot \vec{r}\right){|}_{\theta },$$

and produces the familiar spatial pattern of grid fields, brought about by the constructive interference of the contributing waves (Figure 4B). Next, given the rat's position in the lattice basis, $\vec{r}=m{\vec{e}}_{1}^{g}+n{\vec{e}}_{2}^{g}+\delta \vec{r}$ and using ${\vec{l}}_{3}^{g}={\vec{e}}_{2}^{g}-{\vec{e}}_{1}^{g}$, yields


(19) $$\begin{array}{r} A_{g}(\vec{r})=\cos\frac{2\pi }{3}(2m+n+\delta_{1})\cos\frac{2\pi }{3}(2n+m+\delta_{2})\\ \cos\frac{2\pi }{3}(n-m+\delta_{2}-\delta_{1}){|}_{\theta }, \end{array}$$

where $\delta_{k}=2\delta \vec{r}\cdot {\vec{e}}_{k}^{g}$ are the remainder phases. Curiously, each multiplier in (19) is a discrete-holomorphic constant: the second coincides with (10), the first can be obtained from (10) by re-indexing, *m* ↔ *n*, and the last is produced by an index shift, *n* → *n* − 3*m*. Even more surprisingly, the full product (19), adjusted by a constant reference value 1/4, is also nullified by the discrete Cauchy operators,


$$Q\left(A_{g}-1/4\right)=\bar{Q}\left(A_{g}-1/4\right)=0,$$

which means that the amplitude of grid cells' firing (18) is, in fact, a basic DCA object—a fine-grained discrete-holomorphic constant that functionally highlights the lattice of firing fields. The neurons that respond to grid cell outputs can hence be viewed as functions on that lattice, which includes discrete-holomorphic functions used for modeling border cells. Furthermore, the necessary elements of the DCA can be constructed independently within the oscillatory model, as discussed below.

## 4. Border cells

**Oscillatory model** of the grid cells can be generalized to simulate border cells' activity by replacing the constant membrane potential (17) with suitable discrete-holomorphic functions obeying the maximum principle. The resulting firing rate will then grow toward the boundary of the navigated environment E and produce the characteristic border cell firing patterns.

A simple implementation of this idea can be achieved using the discrete-analytic polynomials (12), by replacing the combinations


$$\theta_{1}=2m+n,\theta_{2}=m+2n,\theta_{3}=n-m,$$

with the phases appearing in (18),


$$\theta_{i}\to \varphi_{i}\equiv \frac{2\pi }{3a_{g}}\langle {\vec{l}}_{i}^{\;g}\cdot \vec{r}\rangle ,$$

that represent dendritic inputs into the postsynaptic cell (Almeida et al., 2009). The resulting fine-grained discrete-holomorphic polynomials are then


(20a) $$P_{1}^{h}=-\frac{\sqrt{3}}{3}\varphi_{12}h_{1}-\frac{1}{2}\varphi_{3}h_{2},$$

(20b) $$P_{2}^{h}=\varphi_{3}(2\varphi_{12}-1)h_{1}-\sqrt{3}\left(\frac{4}{6}\varphi_{12}^{2}-\frac{1}{2}\varphi_{3}^{2}-2\varphi_{12}\right)h_{2},$$

(20c) $$\begin{array}{l} P_{3}^{h}=\varphi_{3}(\varphi_{1}\varphi_{2}-2(2\varphi_{12}-1))h_{1}\\ \;+\sqrt{3}\left(4\varphi_{12}-2\varphi_{12}^{2}+\frac{2}{9}\varphi_{12}^{3}-\frac{1}{2}\varphi_{3}^{2}(\varphi_{12}-1)\right)h_{2}, \end{array}$$

where *φ*_12_ is a short notation for (*φ*_1_ + *φ*_2_)/2 and the waves *h*_1_, *h*_2_ in (12) can be steered along any of the symmetric directions, ${\vec{l}}_{1}$, ${\vec{l}}_{2}$, or ${\vec{l}}_{3}$.

Physiologically, it is possible^5^ that border cell activity is gated by inputs from the grid cells (Katz and Frost, 1996; Floresco and Grace, 2003; Gisiger and Boukadoum, 2011; Hayman and Jeffery, 2008; Giocomo, 2016; Rowland et al., 2018). This mechanism can be modeled by replacing the “undulating" holomorphic constants *h*_1_ and *h*_2_ in (12) with the grid cell firing amplitudes, *A*_*g*_ and the complementary combination of holomorphic sine waves Ã_*g*_ = sin*φ*_1_ sin*φ*_2_ sin*φ*_3_ (Figure 4C), which yields *grid polynomials*, e.g.,


(21a) $$P_{1}^{g}=-\frac{\sqrt{3}}{3}\varphi_{12}A_{g}-\frac{1}{2}\varphi_{3}{\tilde{A}}_{g},$$

(21b) $$\begin{array}{l} P_{2}^{g}=\varphi_{3}(2\varphi_{12}-1)A_{g}-\sqrt{3}\left(\frac{4}{6}\varphi_{12}^{2}-\frac{1}{2}\varphi_{3}^{2}-2\varphi_{12}\right){\tilde{A}}_{g},\\ \text{ etc.,} \end{array}$$

defined explicitly over the grid field lattice (Figure 4D). By direct verification, both sets of polynomials (20) and (21) are discrete-analytic functions that obey the maximum principle and can hence serve as building blocks for producing generic membrane potentials accumulating toward the boundaries of the navigated enclosures.

As mentioned above, the individual *φ*-terms in (20) and (21) may be physiologically interpreted as the inputs received through linear or non-linear synapses. Since the second- and the third-order non-linear synapses are discussed in the literature (Rajan et al., 2013; Rajan and Bialek, 2013; Liu et al., 2022; Latimer et al., 2019; Maheswaranathan et al., 2018; Brivio et al., 2021; Bicknell and Häusser, 2021; Biane et al., 2021; Todo et al., 2019; Wang and Dudko, 2021; Rossbroich et al., 2021), we used combinations of 5–10 polynomials of the orders *r*_*i*_ = 1, 2, 3,


(22) $$\mu_{b}={\left[\alpha_{1}P_{r_{1}}^{*}+\alpha_{2}P_{r_{2}}^{*}+\ldots +\alpha_{q}P_{r_{q}}^{*}\right]}_{\theta }.$$

**Figure 5:**
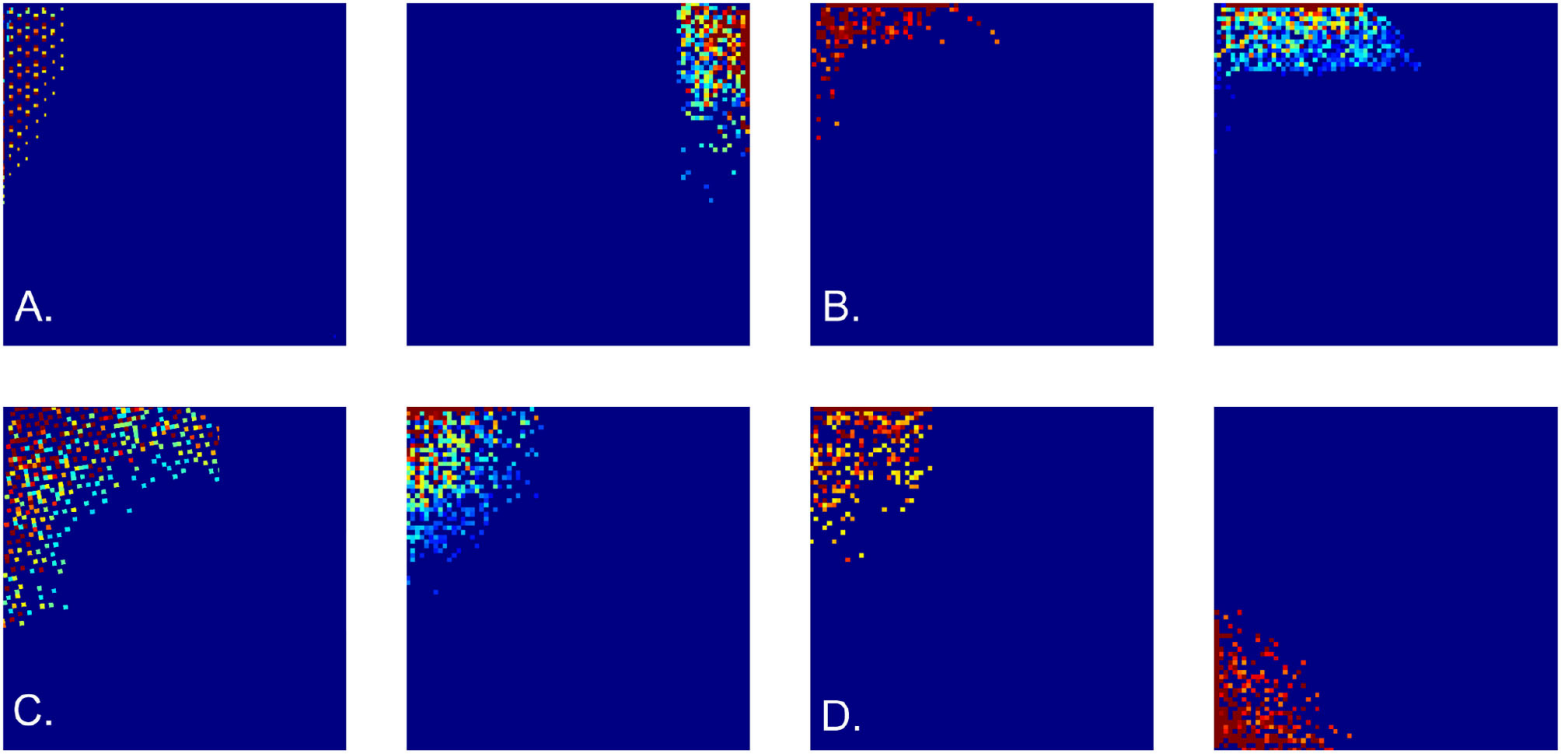
Cell firing patterns. Examples of simulated border cell firing fields in a square 6 × 6 m environment, obtained as combinations of first, second, and third order grid polynomials. **(A)** Firing fields obtained using “undulating" discrete-holomorphic polynomials (12). **(B)** Examples of the firing fields obtained using combinations of grid-holomorphic polynomials (21). **(C)** Firing fields of noise-perturbed membrane potentials, for ε = 0.2 (left panel) and ε = 0.3 (right panel). **(D)** Firing fields obtained using the schematic network model.

Here the $P_{r}^{*}$ represent either harmonic (20) or the grid polynomials (21), the coefficients α_*i*_ define the magnitude of each addend, and the θ subscript indicates the threshold. In the simulations, the values α_*i*_ were selected randomly, while the threshold grew according to the size of the environment and the order of the contributing polynomials, $\theta \propto {\left(L/a_{g}\right)}^{r_{i}}$. The resulting firing maps are illustrated on Figures 5A, B. Expectedly, since all contributing polynomials in (22) grow toward the boundaries of the available lattice domain, all simulated border cells fire along the frontiers of the navigated enclosure.

Importantly, these outcomes are robust with respect to stochastic variations: disturbing the phases *φ*_*i*_ of the holomorphic polynomials with a noise term, εξ, where ξ is a random variable uniformly distributed over [0, 2π] and ε controls its amplitude, does not qualitatively alter the resulting spatial patterns for ε ≤ 0.5 or more (Figure 5C).

**Schematic network model**. Defining the membrane potentials as functions of speed and coordinates used, e.g., in (17) helps linking the geometry of the observed environment to the underlying neuronal computations. However, modeling the brain's own representation of the ambient environment requires using intrinsic representation of spatial information, a key role in which is played by hippocampal place cells, *c*_*i*_, and the postsubicular^6^ head direction cells, *h*_*i*_ (Grieves and Jeffery, 2017; Taube, 1998). The computational units enabling this representation are the functionally interconnected cell groups


(23σ) $$\sigma_{i}=[c_{i_{0}},c_{i_{1}},\ldots ,c_{i_{n}}],$$

(23η) $$\eta_{j}=[h_{j_{1}},h_{j_{2}},\ldots ,h_{j_{n}}],$$

which highlight, respectively, basic locations υ_σ_*i*__ and angular domains υ_η_*j*__ (Harris, 2005; Buzsáki et al., 2014; Peyrache et al., 2015; Brandon et al., 2013; Maurer et al., 2006). A number of studies have demonstrated that the assemblies (23) encode the animal's ongoing position, the shape of trajectory and even its planned and recalled navigational routes (Brown et al., 1998; Frank et al., 2000; Guger et al., 2011; Karlsson and Frank, 2009; Johnson and Redish, 2007; Dragoi and Tonegawa, 2011; Pfeiffer and Foster, 2013). By the same principle, place cell assemblies that fire over the grid fields υ_*g*_*i*__, can provide their hippocampal representation: a combination $\hat{\sigma_{i}}$ of σ-assemblies whose constituent cells exhibit coactivity with a grid cell *g* and each other defines a vertex of grid cell activity,


(24) $${\hat{v}}_{i}^{g}=[{\hat{\sigma }}_{i},g].$$

In the following, the superscript “*g*" will be suppressed in describing single grid cell activity and used only to distinguish contributions from different grid cells.

The hexagonal order on the vertexes (24) is established by concomitant activity of select groups of head direction assemblies, ${\hat{\eta }}_{1},{\hat{\eta }}_{2},\ldots ,{\hat{\eta }}_{6}$, that activate on the runs between pairs of neighboring grid fields, e.g., υ_*i*_ and υ_*j*_, thus defining the *spiking edges* between ${\hat{\text{v}}}_{i}$ and ${\hat{\text{v}}}_{j}$,


(25) $$\epsilon_{ij}^{k}=\{{\hat{\sigma }}_{i},{\hat{\sigma }}_{j}|{\hat{\eta }}_{k},g\}.$$

Together, the vertexes (24) and the edges (25) can be viewed as elements of a *spike-lattice* $V_{g}$, by which the grid field lattice is embedded in the cognitive map (Dabaghian, 2023). Using $V_{g}$ allows constructing a self-contained phenomenological network model of border cells that does not involve “tagging" the neuronal activity by externally observed characteristics, such as the rat's speed or Euclidean coordinates.

Suppose that a cell *b* with membrane potential μ_*b*_ receives input from a group of persistently firing head direction assemblies ${\hat{\eta }}_{k}$, over a period when grid cell *g* becomes active, then shuts down, and then restarts its activity again.^7^ If these consecutive activations are induced over adjacent vertexes ${\hat{\text{v}}}_{i}$ and ${\hat{\text{v}}}_{j}$, then the corresponding change of the membrane potential can be interpreted as the change of the spike-lattice function $\mu_{b}\left(\hat{\text{v}}\right)$ along the edge ϵ_*ij*_ between them,


(26) $$\begin{array}{l} \left[{\hat{\sigma }}_{i},{\hat{\sigma }}_{j},{\hat{\eta }}_{k},g\right]⇝\mu_{b}\left({\hat{\text{v}}}_{i}\right)=\mu_{b}\left({\hat{\text{v}}}_{j}\right). \end{array}$$

On the other hand, the transformation (26) can be described as the action of a *spike-lattice shift operator* $\hat{\tau }$ on μ_*b*_,


$$\begin{array}{l} \hat{\tau }\mu_{b}\left({\hat{\text{v}}}_{i}\right)=\mu_{b}\left({\hat{\text{v}}}_{j}\right). \end{array}$$

In particular, changes induced by the head direction assemblies ${\hat{\eta }}_{1}$ and ${\hat{\eta }}_{2}$ (ordered as on Figure 2A) can be identified with the shift operators acting “forward” along the basic lattice directions,


(27a) $$\begin{array}{l} {\hat{\tau }}_{1}\mu_{b}\left(\hat{\text{v}}\right)=\mu_{b}\left({\hat{\text{v}}}_{+}^{\prime }\right)\text{ and }{\hat{\tau }}_{2}\mu_{b}\left(\hat{\text{v}}\right)=\mu_{b}\left({\hat{\text{v}}}_{+}^{\prime \prime }\right), \end{array}$$

while the “opposite” assemblies ${\hat{\eta }}_{4}$ and ${\hat{\eta }}_{5}$ induce backward transformation,


(27b) $$\begin{array}{l} {\hat{\tau }}_{1}^{-1}\mu_{b}\left(\hat{\text{v}}\right)=\mu_{b}\left({\hat{\text{v}}}_{-}^{\prime }\right)\text{ and }{\hat{\tau }}_{2}^{-1}\mu_{b}\left(\hat{\text{v}}\right)=\mu_{b}\left({\hat{\text{v}}}_{-}^{\prime \prime }\right). \end{array}$$

The appearance of spiking analogues of the shift operators *τ*_1_ and *τ*_2_ associated with grid cells opens a possibility of implementing the key DCA structures neuronally. However, a principal challenge in this approach is that the series of inputs received along a particular trajectory may not concur with the lattice structure of the underlying grid fields. Indeed, consider the membrane potential at the initial spiking vertex ${\hat{\text{v}}}_{0}$,


$$\begin{array}{l} \mu_{b}\left({\hat{\text{v}}}_{0}\right)=U\left({\hat{\text{v}}}_{0}\right)A_{g}\left({\hat{\text{v}}}_{0}\right)+W\left({\hat{\text{v}}}_{0}\right){Ã}_{g}\left({\hat{\text{v}}}_{0}\right), \end{array}$$

from where the animal continues to move along a trajectory γ, producing a series of postsynaptic changes described by a sequence of $\hat{\tau }$-shifts,


(28) $$\begin{array}{l} \mu_{b}\left({\hat{\text{v}}}_{f}\right)={\hat{\tau }}_{i_{1}}{\hat{\tau }}_{i_{2}}\ldots {\hat{\tau }}_{i_{k}}\cdot \left(U\left({\hat{\text{v}}}_{0}\right)A_{g}\left({\hat{\text{v}}}_{0}\right)+W\left({\hat{\text{v}}}_{0}\right){Ã}_{g}\left({\hat{\text{v}}}_{0}\right)\right). \end{array}$$

If the net membrane potential (28) does not depend on the order in which the individual inputs arrive, the $\hat{\tau }$-operators commute.^8^ Thus, the value accrued at the final vertex ${\hat{\text{v}}}_{f}$ is


(29) $$\begin{array}{l} \mu_{b}\left({\hat{\text{v}}}_{f}\right)\equiv \mu_{b}\left({\hat{\text{v}}}_{\tilde{m},ñ}\right)={\hat{\tau }}_{1}^{\tilde{m}}{\hat{\tau }}_{2}^{ñ}\cdot \left(U\left({\hat{\text{v}}}_{0}\right)A_{g}\left({\hat{\text{v}}}_{0}\right)+W\left({\hat{\text{v}}}_{0}\right){Ã}_{g}\left({\hat{\text{v}}}_{0}\right)\right), \end{array}$$

where the integers $\tilde{m}$ and ñ mark how many times ${\hat{\tau }}_{1}^{\pm }$ and ${\hat{\tau }}_{2}^{\pm }$ were triggered along the way.

**Figure 6:**
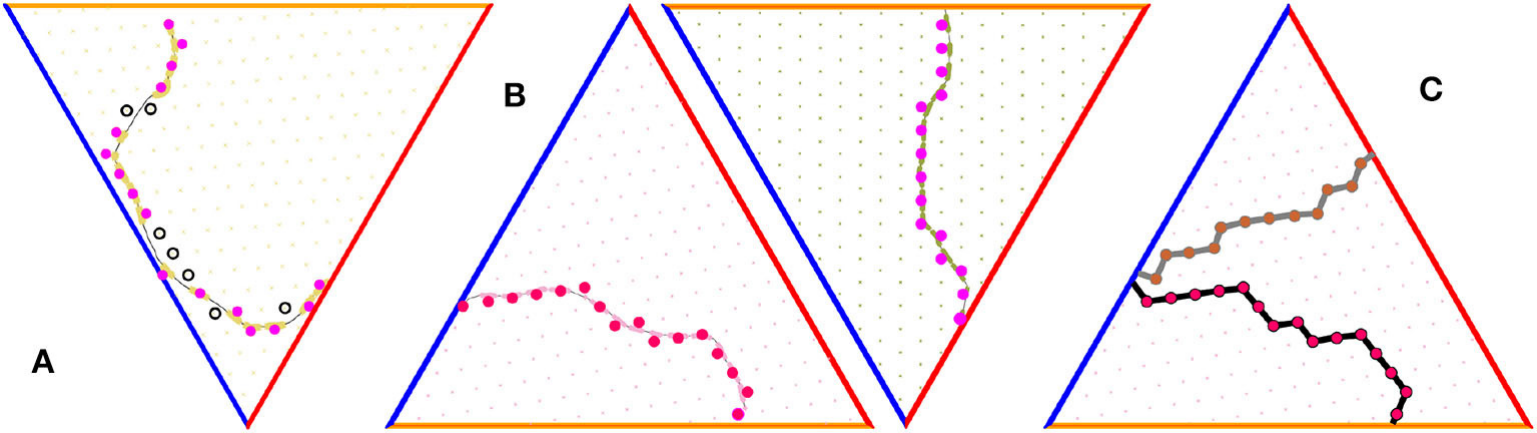
Grid cell percolation and border cell firing patterns. **(A)** Generic path is non-percolative: the vertexes that correspond to the “percolated" firing fields—the ones over which the grid cell has produced at least one spike—are marked by pink, while vertexes corresponding to the fields that did not respond are marked by black. **(B)** Two examples of percolating trajectories, along which the spiking occurred at each vertex, without omissions. **(C)** Two examples of lattice paths induced by two percolating trajectories.

Note however, that a generic trajectory γ may not pass through the fields of a given cell *g* in complete sequence: some fields are visited, others are occasionally missed (Figure 6A). As a result, the “empirical" $\left(\tilde{m},ñ\right)$-indexing appearing in (29) may not conform with the original (*m, n*)-indexing of the full grid field set, which moots the possibility of interpreting the argument of μ_*b*_ in terms of the underlying lattice (14). However, it can be shown that, within physiological parameter range, there typically exists a special class of “percolating" paths—those that run through the firing fields of a given grid cell in contiguous sequence, without omissions (see Dabaghian, 2023, Figure 6B). Such paths induce series of conjoint spiking edges,


(30) $$\begin{array}{l} 𝔊\left(\gamma \right)\equiv \left\{\epsilon_{ij},\epsilon_{jk},\ldots ,\epsilon_{pq}\right\}, \end{array}$$

that serve as lattice representations of the animals' moves (Figure 6C, Dabaghian, 2023). The increments of the postsynaptic membrane potential (29) acquired along the link series (30) are, by design, compatible with the lattice indexing and hence allow constructing consistent lattice functions over an extended lattice domains (Dabaghian, 2023). The subsequent development of the model will therefore be based on percolating paths only.

Constructing a membrane potential (29) by applying spiking $\hat{\tau }$-operators along the percolated paths requires knowing how these operators act on discrete-holomorphic constants and polynomials, which can be established as follows. First, the response of the spike-lattice counterparts of holomorphic constants *h*_1_, *h*_2_, and of their grid analogues, $A_{g}\left(\hat{\text{v}}\right)$ and ${Ã}_{g}\left(\hat{\text{v}}\right)$, to $\hat{\tau }$-shifts (27), can be implemented according to how the corresponding original, index-dependent expressions (11) and (19) respond to the *τ*-operators, e.g.,


(31.1) $$\begin{array}{l} {\hat{\tau }}_{1}^{\pm 1}A_{g}\left(\hat{\text{v}}\right)=-\frac{1}{2}A_{g}\left(\hat{\text{v}}\right)\pm \frac{\sqrt{3}}{2}{Ã}_{g}\left(\hat{\text{v}}\right), \end{array}$$

(31.2) $$\begin{array}{l} {\hat{\tau }}_{2}^{\pm 1}{Ã}_{g}\left(\hat{\text{v}}\right)=-\frac{1}{2}{Ã}_{g}\left(\hat{\text{v}}\right)\mp \frac{\sqrt{3}}{2}A_{g}\left(\hat{\text{v}}\right). \end{array}$$

One can then use the expressions (31) along with (27) as the rules defining how the $\hat{\tau }$s act on the spike-lattice $V_{g}$, and thus deduce how the “spiking" Cauchy operator $\bar{Q}$ acts on generic membrane potentials,


(32) $$\begin{array}{r} \bar{Q}\mu_{b}(\hat{v})=(U(\hat{v})-\frac{1}{2}U({\hat{v}}_{-}^{\prime })-\frac{1}{2}U({\hat{v}}_{-}^{\prime \prime })\\ +\frac{\sqrt{3}}{2}(W({\hat{v}}_{-}^{\prime })-W({\hat{v}}_{-}^{\prime \prime })))A_{g}(\hat{v})\\ +(W(\hat{v})-\frac{1}{2}W({\hat{v}}_{-}^{\prime })-\frac{1}{2}W({\hat{v}}_{-}^{\prime \prime })\\ +\frac{\sqrt{3}}{2}(U({\hat{v}}_{-}^{\prime })-U({\hat{v}}_{-}^{\prime \prime }))){\tilde{A}}_{g}(\hat{v}). \end{array}$$

To satisfy the discrete analyticity condition, $\bar{Q}\mu_{b}\left(\hat{\text{v}}\right)=0$, the coefficients in front of the holomorphic constants in (32) must vanish at each spike-vertex $\hat{\text{v}}$. The simplest solution to this requirement is provided by the functions that acquire constant increments over the vertex shifts,


(33u) $$\begin{array}{l} U\left({\hat{\text{v}}}_{\pm }^{\prime }\right)=U\left(\hat{\text{v}}\right)\pm C_{1},U\left({\hat{\text{v}}}_{\pm }^{\prime \prime }\right)=U\left(\hat{\text{v}}\right)\pm C_{2}, \end{array}$$

(33w) $$\begin{array}{l} W\left({\hat{\text{v}}}_{\pm }^{\prime }\right)=W\left(\hat{\text{v}}\right)\pm D_{1},W\left({\hat{\text{v}}}_{\pm }^{\prime \prime }\right)=W\left(\hat{\text{v}}\right)\pm D_{2}. \end{array}$$

By direct verification, the equation $\bar{Q}\mu_{b}\left(\hat{\text{v}}\right)=0$ is satisfied identically if


(34) $$\begin{array}{l} C_{2}=-C_{1}=C,D_{1}=D_{2}=\sqrt{3}C, \end{array}$$

where *C* represents vertex-independent additive synaptic input. Thus, if the specific synaptic responses to each of the *τ*s are defined by (34), then the net accumulated postsynaptic membrane potential is


(35) $$\begin{array}{l} \mu_{b}=C\left(m-n\right){Ã}_{g}-C\sqrt{3}\left(m+n\right)A_{g}, \end{array}$$

which matches the linear discrete-holomorphic polynomial (21a) and clarifies how such potential may emerge through synaptic integration. For the non-linear membrane potentials described by higher-order polynomials, the shifting rules can be obtained by analogy with (33), by requiring that the shifted values are described by lower-order polynomials, e.g., by linear increments to the shifted second-order polynomials,


(36u) $$\begin{array}{l} \Delta U_{2}\left(\hat{\text{v}}\right)=U_{1}\left(\hat{\text{v}}\right)+C_{1}^{\prime },\Delta U_{2}\left(\hat{\text{v}}\right)=U_{1}\left(\hat{\text{v}}\right)+C_{2}^{\prime }, \end{array}$$

(36w) $$\begin{array}{l} \Delta W_{2}\left(\hat{\text{v}}\right)=W_{1}\left(\hat{\text{v}}\right)+D_{1}^{\prime },\Delta W_{2}\left(\hat{\text{v}}\right)=U_{1}\left(\hat{\text{v}}\right)+D_{2}^{\prime }, \end{array}$$

and so forth. The results then produce second and third order expressions of the type (20b) and (21*b*), which combine according to (22) and yield build border cell firing patterns as illustrated on Figure 5D.

## 5. Discussion

A number of computational models aim to explain the origins of the triangular spatial pattern of the grid cells' spiking activity and the contribution that these cells make into enabling spatial cognition (Giocomo et al., 2011). It is believed that the regular grid firing patterns allow establishing global metric scale in the navigated environment (Moser et al., 2008) and may produce a spatial location code (Welinder et al., 2008; Sreenivasan and Fiete, 2011; Burak and Fiete, 2012). The model discussed above shows that the neuronal mechanisms producing hexagonal layout of the firing fields may enable yet another mathematical phenomenon—a discrete complex structure. Although the whole structure is implemented via real-valued computations, it captures all the key attributes of the conventional theory of complex variables. In particular, the discrete-analytic functions defined in DCA framework obey the maximum principle—a property that may be used to model neurons with firing responses tuned to the boundaries of the navigated environments.

Surprisingly, basic elements of DCA appear implicitly in a few existing models of the grid cells. As discussed above, the interfering waves of the oscillatory models, which may be viewed either as representations of physiological rhythms, such as extracellular or submembrane potential oscillations, or as formal components of the membrane potential's spatiotemporal decomposition, can be interpreted as spatially fine-grained discrete holomorphic constants. Their interference pattern, that defines the grid cells' firing amplitude (17), also produces a discrete-holomorphic “grid” constant (19), that highlights a triangular lattice.

From the perspective of DCA, this construction admits a natural generalization, based on replacing zeroth order constants with higher-order polynomials (and hence generic discrete-holomorphic functions), which yields firing patterns characteristic for border cells. Indeed, if a cell's membrane potential arises from a combination of discrete-holomorphic lineals, quadratics, cubics, etc. (Equations 20–22), then the corresponding spiking is boundary-bound, by virtue of the maximum principle.

The existence of a common framework for describing the grid and the border cells points at their physiological affinity: potentially, different neurons may implement the same spiking mechanism, outlined above, but involve synaptic integrations of different orders, and thus yield either grid-like or border-preferring firing activity. Furthermore, such neurons may, conceivably, swap their firing profiles through synaptic or structural plasticity changes. The latter may explain why these cells are anatomically intermingled—in electrophysiological recordings, both cell types are often detected on the same tetrode.

Although physiological validity of the oscillatory interference model is debated (Barry et al., 2012), its key elements, e.g., θ-modulation of the membrane potential and spike times, speed modulation of θ-frequencies, the connection of the latter and the grid scale, *a*_*g*_, etc., were experimentally identified (Jeewajee et al., 2008; Giocomo et al., 2011; Burgess and O'Keefe, 2011; Domnisoru et al., 2013). Validating the additional mechanisms, responsible for the border-bound firing, may then focus on testing whether the membrane potential dynamics over the percolated paths follows the rules (33, 36), thus implementing the DCA principles. A generalization of the DCA outlined in Dynnikov (2015) also suggests that border-bound and grid-like layouts of firing fields may also be associated with generic, not just triangular lattices.

The DCA approach can be also be used to produce self-contained network models that do not require phenomenological inputs, i.e., do not reference speed, coordinates, grid field positions, *ad hoc* lattice indexes (*m, n*) or other externally observed tags of neuronal activity. On the contrary, it becomes possible to render certain abstract DCA structures via autonomous network computations. For example, the Cauchy operators and the lattice (14) underlying the grid field layouts are induced using the “spiking" analogues of the *τ*-operators (4),


(37) $$\begin{array}{l} V_{g}=\left\{{\hat{\text{v}}}_{m,n}=m{\hat{\tau }}_{1}+n{\hat{\tau }}_{2},m,n\in \mathbb{Z}\right\}, \end{array}$$

with vertex indexes derived from counting synaptic inputs of the grid, head direction and place cells along the percolated paths. In this context, the standard procedure of constructing grid fields $\upsilon_{m,n}^{g}$ (Figure 1C), by attributing (*x*_1_, *x*_2_) coordinates to spikes according to the rat's ongoing location, can be viewed as a mapping from the vertexes of the spike lattice (37) into regions in the navigated environment,


$$\begin{array}{l} f_{g}:{\hat{\text{v}}}_{m,n}^{g}\to \upsilon_{m,n}^{g}\in E, \end{array}$$

centered at the vertexes of the grid field lattice *V*_*g*_ (Dabaghian, 2022; Babichev et al., 2016). Zero holonomy property of the discrete Cauchy operators discussed in Novikov and Dynnikov (2003); Novikov (2004) (see also Dabaghian, 2016) ensures that the (*m, n*) values attained at a particular vertex do not depend on the percolating paths leading to a vertex, but only on the vertex itself, which ensures consistency of the construction. The discrete-complex structure can thus be viewed as an intrinsic network property, that may be implemented using different synaptic architectures, e.g., the continuous attractor models. An implication of this property is that the grid cells should be expected to produce planar, rather than voluminous firing fields, in order to implement the Cauchy decomposition (7) attainable only on 2*D* hexagonal lattices—a prediction that agrees with both experimental (Hayman et al., 2011, 2015; Soman et al., 2018; Ginosar et al., 2021; Grieves et al., 2021) and theoretical (Horiuchi and Moss, 2015; Mathis et al., 2015; Stella and Treves, 2015; Gong and Yu, 2021) studies.

As a concluding comment, the DCA framework currently does not offer a direct geometric interpretation of the discrete-holomorphic mappings (Novikov and Dynnikov, 2003; Novikov, 2004, 2011). An independently developed notion of discrete conformal transformations, based on rearrangements of regular circle packings in planar domains (Köbe, 1936; Thurston, 1985; Rodin and Sullivan, 1987; Bücking, 2008) may therefore offer a complementary venue for establishing correspondences between network activity and discrete-complexity. Several recent experimental (Savelli et al., 2008; Zhang et al., 2014; Krupic et al., 2016, 2018; Savelli et al., 2017; Wernle et al., 2018; Bellmund et al., 2020) and theoretical (Urdapilleta et al., 2015; Santos-Pata et al., 2017; Spalla et al., 2019; Monsalve-Mercado and Leibold, 2020; Zhang et al., 2023) studies suggest that conformal transformations of the navigated spaces may induce compensatory discrete-conformal transformations of the grid field maps, similar to how the hippocampal place cells tend to preserve coactivity patterns in morphing environments (Gothard et al., 1996; Dabaghian et al., 2014; Rueckemann et al., 2021). If the latter is verified experimentally, it can be argued that the grid cell inputs constrain the hippocampal topological map (Dabaghian et al., 2014; Rueckemann et al., 2021), to a net conformal map of the navigated space.

## Data availability statement

The original contributions presented in the study are included in the article/supplementary material, further inquiries can be directed to the corresponding author.

## Author contributions

The author confirms being the sole contributor of this work and has approved it for publication.

## Footnotes

^1^Throughout the text, terminological definitions and semantic highlights are given in *italics*.

^2^DCA can also be constructed over the complex numbers: the corresponding theory then yields the standard Complex Analysis in the limit when the lattice side vanishes (Novikov and Dynnikov, 2003; Novikov, 2004, 2011; Dynnikov, 2015).

^3^The operators *Q* and $\bar{Q}$ generally do not distribute according to the Leibniz rile, e.g.,

$$Q\left(f\left(m,n\right)h_{2}\right)\neq \left(Qf\left(m,n\right)\right)h_{1}+f\left(m,n\right)\left(Qh_{2}\right).$$

^4^The model (Fuhs and Touretzky, 2006) uses sum of three waves for capturing analogous interference effect.

^5^Currently, the synaptic organization of the border cell network is debated.

^6^Head direction cells are also found in few other brain regions (Taube, 1998).

^7^For a physiological discussion, see Hasselmo (2008a,b); Egorov et al. (2002); Dabaghian (2023).

^8^As do their *τ*-counterparts (Novikov and Dynnikov, 2003; Novikov, 2004, 2011; Dynnikov, 2015).
